# Reported selection criteria for adult acquired flatfoot deformity and posterior tibial tendon dysfunction: Are they one and the same? A systematic review

**DOI:** 10.1371/journal.pone.0187201

**Published:** 2017-12-01

**Authors:** Megan H. Ross, Michelle D. Smith, Bill Vicenzino

**Affiliations:** School of Health and Rehabilitation Sciences, University of Queensland, Brisbane, Australia; University of Illinois at Urbana-Champaign, UNITED STATES

## Abstract

**Background:**

Posterior tibial tendon dysfunction (PTTD) and adult acquired flatfoot deformity (AAFD) are used interchangeably, although both suggest quite different pathological processes.

**Objective:**

To investigate key differences in selection criteria used for inclusion into research studies.

**Methods:**

An electronic database search was performed from inception to June 2016. All primary research articles with clear inclusion/diagnostic criteria for PTTD or AAFD were included in the review. All criteria were extracted and synthesised into one aggregate list. Frequencies of recurring criteria were calculated and reported for each stage of the conditions.

**Results:**

Of the potentially eligible papers, 148 (65%) did not specify inclusion/selection criteria for PTTD or AAFD and were excluded. Eligibility criteria were reported 82 times in the 80 included papers, with 69 descriptions for PTTD and 13 for AAFD. After synthesis of criteria from all papers, there were 18 key signs and symptoms. Signs and symptoms were considered to be those relating to tendon pathology and those relating to structural deformity. The total number of individual inclusion/diagnostic criteria ranged from 2 to 9. The majority of articles required signs of both tendon dysfunction and structural deformity (84% for AAFD and 81% for PTTD). Across both groups, the most frequently reported criteria were abduction of the forefoot (11.5% of total criteria used), the presence of a flexible deformity (10.2%) and difficulty performing a single leg heel raise (10.0%). This was largely the case for the PTTD articles, whereas the AAFD articles were more focused on postural issues such as forefoot abduction, medial arch collapse, and hindfoot valgus (each 16.7%).

**Conclusion:**

As well as synthesising the available literature and providing reporting recommendations, this review has identified that many papers investigating PTTD/AAFD do not state condition-specific selection criteria and that this limits their clinical applicability. Key signs and symptoms of PTTD and AAFD appear similar, except in early PTTD where no structural deformity is present. We recommend that PTTD is the preferred terminology for the condition associated with signs of local tendon dysfunction with pain and/or swelling along the tendon and difficulty with inversion and/or single leg heel raise characterising stage I and difficulty with single leg heel raise and a flexible flatfoot deformity characterizing stage II PTTD. While AAFD may be useful as an umbrella term for acquired flatfoot deformities, the specific associated aetiology should be reported in studies to aid consolidation and implementation of research into practice.

**Trial registration:**

Prospero ID: 42016046943

## Background

Presentation of a progressively flat foot with medial ankle pain is likely to be diagnosed as a posterior tibial tendon dysfunction (PTTD) or an adult acquired flatfoot deformity (AAFD) [1, 2]. These terms seem to be used interchangeably in the literature [3–5], even though they suggest possible dysfunction of different structures. The evolution of the terminology used for this condition began with emphasizing the tendon pathology; PTTD [6–8] and increasingly over recent times the focus has shifted to the foot deformity; AAFD. The characteristic flat foot deformity, and the notion that PTTD does not adequately describe the ligamentous failure and resultant joint destruction that ultimately occurs [3, 9], are possible reasons for the adoption of AAFD terminology. The problem with using the term AAFD is that in addition to being a result of PTTD [6, 10], it also results from other aetiologies, such as traumatic (injury to ligament or tendon), degenerative, arthritic and neuromuscular conditions [11–14]. This situation is potentially problematic in both clinical practice and research, because AAFD may not adequately represent the underlying pathology and consequently the diagnosis. One of the problems with this is that management decisions are likely to differ according to the diagnosis. This review will systematically synthesise the key signs and symptoms of PTTD and AAFD from the literature to ascertain if there is a difference in diagnostic criteria related to nomenclature and provide recommendations for selection criteria to be used in future research.

## Methods

A systematic review of the literature reporting work on PTTD and AAFD was undertaken to test the hypothesis that there would be overlapping terminology for selection criteria used by investigators in PTTD and AAFD literature.

### Search strategy

Electronic databases (CINAHL, Cochrane, Embase, PubMed and Web of Science) were comprehensively searched by one reviewer (MR) for all years available up to and including June 13 2016. The search strategy was developed in consultation with an experienced academic librarian and was undertaken using a combination of keywords and MeSH terms. Keywords used in the search strategy aimed to capture all past and present variations in terminology for the condition: Flatfoot OR (posterior AND tibia* AND (tendon* OR tendin*)) OR “pes planus” OR “pes planovalgus”. No restrictions or second string limitations were used to further narrow the search. All search results were imported in Endnote X7 (Thompson Reuters, Philadelphia, PA, USA) and duplicates were removed. This systematic review followed the Preferred Reporting Items for Systematic Reviews and Meta-Analyses (PRISMA) guidelines and it was registered at http://www.crd.york.ac.uk/PROSPERO/display_record.asp?ID=CRD42016046943.

### Selection criteria

Articles were included if they investigated PTTD or AAFD and clearly defined diagnostic or inclusion criteria. Articles were excluded if they did not investigate PTTD or AAFD, and did not detail specific inclusion or diagnostic criteria (i.e. reported that diagnosis was only made by a specified health professional or was only based on a PTTD or AAFD classification system without detailing specific clinical signs or symptoms). Asymptomatic flatfoot conditions were not considered for this review. Non-English publications, clinical reviews/narratives or single case reports, as well as paediatric, animal and cadaveric studies were also excluded. Assessment of study eligibility was performed by one investigator (MR) and uncertainties were resolved by two other reviewers (BV and MS).

### Data extraction and synthesis

A custom data extraction table was developed. One reviewer (MR) extracted data from all included studies including condition (PTTD or AAFD), stage of condition (I–IV; based on classification systems) and individual inclusion or diagnostic criteria specified in the article. Studies included in this review staged the condition using the Johnson & Strom [8], Myerson [15] and Bluman [16] classification systems. The Johnson & Strom and Myerson classification systems are the same and hence forth referred to as the former. It consists of 4 progressive stages [8, 15] defined as follows: I) tenosynovitis and mild to moderate pain and tenderness of the posterior tibial tendon, with no signs of foot deformity; II) degeneration and elongation of the tendon and flexible hindfoot eversion with forefoot abduction; III) rigid hindfoot eversion with forefoot abduction; IV) the same as III) with valgus angulation of the talus and degeneration of the tibiotalar joint [15]. The classification system defined by Bluman maintains the existing outline of Johnson & Strom, except each stage is divided into sub-categories, which include reference to radiographic findings and more refined delineation of presenting signs and symptoms [16]. For example, Bluman’s Stage IIB refers to talonavicular uncovering on radiographs, as well as flexible hindfoot eversion with forefoot abduction [16]. In this review the specific classification system used in each paper is indicated by the format used (i.e IIB indicates Bluman classification system was used, whereas II indicates Johnson & Strom was used).

All criteria presented in individual papers were initially recorded using the exact terminology from the study (S1 Table) and then reduced to key terms for reporting (e.g., hindfoot valgus was used as a key term to represent heel valgus, calcaneal valgus, hindfoot eversion). These key terms formed the aggregate list of diagnostic/inclusion criteria against which all included studies were scored.

We sought to represent criteria used in each paper by categorising them as either being compulsory (mandatory signs or symptoms required for diagnosis or inclusion) or optional (one of a number of possible signs or symptoms required for diagnosis or inclusion). When signs and symptoms were listed with the conjunction “and”, all criteria were considered to be compulsory. Where criteria were listed with “or” as the conjunction, or “at least one of” preceding the criteria, each criteria was considered to be optional, but the group of optional criteria (with ‘or’ ‘at least one of’ operand) was considered as one compulsory criterion. The total number of criteria for each description of diagnostic/inclusion criteria was the total number of compulsory (single or grouped optional) criteria.

## Results

**Fig 1:**
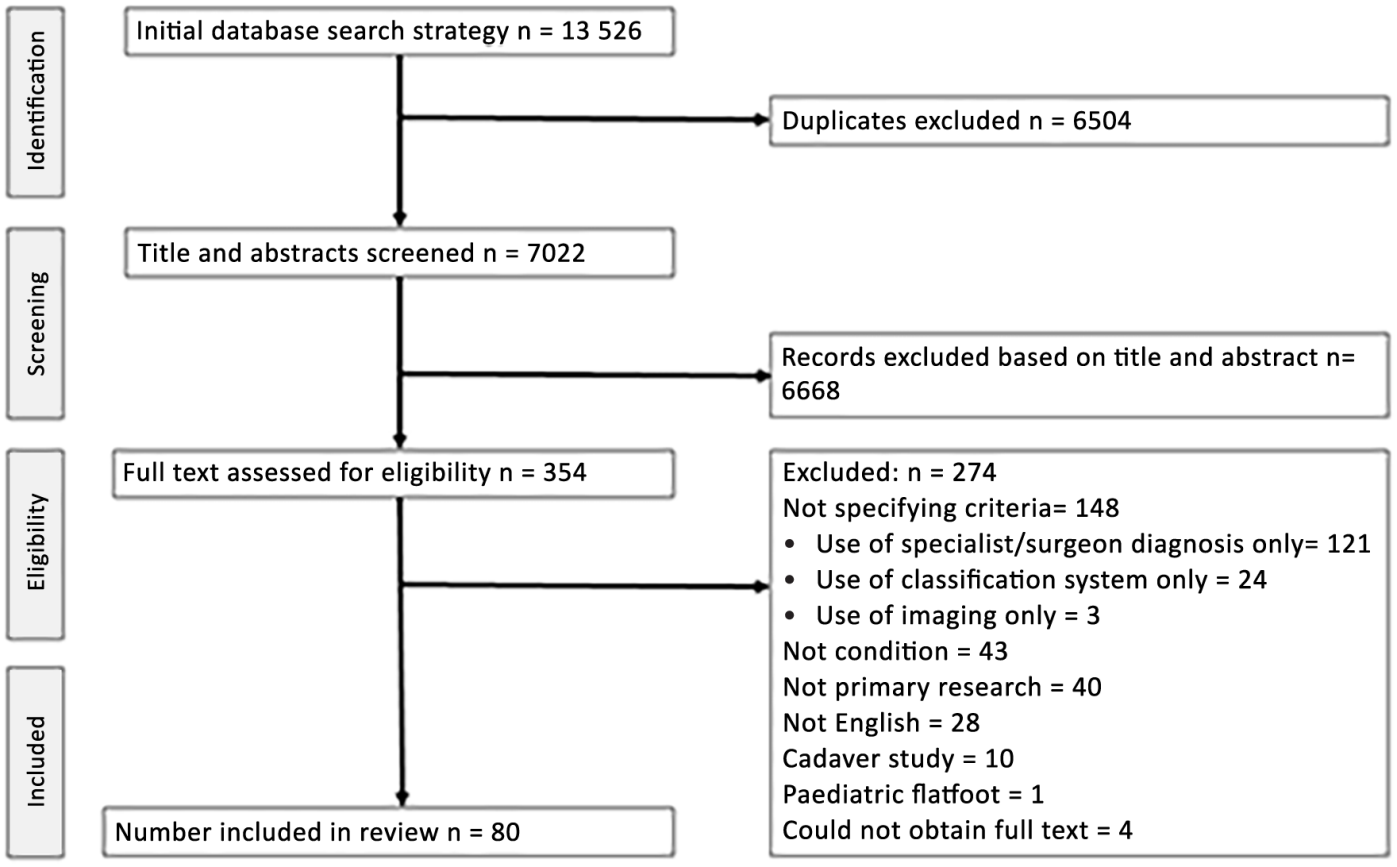
Flow chart of study selection process.

The electronic database search yielded a total of 13 526 records. Fig 1 outlines studies excluded at each stage of the selection process. After screening of title and abstract of all retrieved articles, 354 full text articles investigating either PTTD or AAFD were examined for final inclusion. Following this final full text screening, 80 articles met all inclusion criteria and were included in the review. Diagnostic or inclusion criteria for PTTD or AAFD were specified 82 times in 80 papers (Table 1). One article (Kohls-Gatzoulis, 2009) detailed diagnostic criteria for stage I PTTD, stage II PTTD and AAFD. Sixty-nine of the 82 definitions in the articles were for PTTD and the remaining 13 defined AAFD.

**Table 1:** Selection criteria for posterior tibial tendon dysfunction (PTTD) and adult acquired flatfoot deformity (AAFD) for included studies.

Author	Year	Stage	RELATED TO TENDON DYSFUNCTION										RELATED TO STRUCTURAL DEFORMITY								No. of criteria
			Pain PTT	Pain medial ankle/ foot	TOP PTT	Pain res. INV	Pain with SLHR	Swelling PTT	Swelling medial ankle/ foot	INV strength deficit	Difficulty with SLHR	Dec. walking ability	FF def.	HF valgus	Medial arch collapse	FF ABD	MF ABD	FF SUP	Flex. def.	Talar head promi-nence	
**PTTD**																					
Teasdall [17]	1994	I		1	1						1										**3**
Crates [18]	1999	I			1			1								1					**3**
Perry [19]	2003	I			1	1	1												1		**4**
Sharma [20]	2003	I		1					1		1										**3**
Rosenfeld [21]	2005	I			1					1			1			1			1		**5**
Cooper [22]	2007	I	1					1													**2**
Kohls-Gatzoulis [23]	2009	I				1				1	1										**4**
Rabbito [24]	2011	I		1	1				1												**3**
Chow [25]	2015	I	1					1		1	1										**4**
Hua [26]	2015	I		1	1	1				1											**4**
Chen [27]	1997	II		1	1		1		1												**4**
Hintermann [28]	1999	II		1						1	1		1	1	1	1	1		1		**9**
Toolan [29]	1999	II		1						1				1		1			1		**5**
Conti [30]	2002	II		1						1	1	1	1	1	1	1			1		**9**
Fayazi [31]	2002	II		1						1		1	1	1	1	1			1		**8**
Wacker [32]	2002	II		1					1	1	1		1			1			1		**7**
Viladot [33]	2003	II		1							1		1			1			1		**5**
Wacker [34]	2003	II		1					1	1	1		1			1			1		**7**
Brodsky [35]	2004	II	1		1			1		1	1										**5**
Myerson [36]	2004	II	1		1					1	1			1		1	1		1		**8**
Valderrabano [37]	2004	II		1						1	1	1	1	1	1	1			1		**9**
Needleman [38]	2006	II								1			1						1		**3**
Tome [39]	2006	II																	1		**3**
Knupp [40]	2007	II		1						1	1	1		1				1	1		**7**
Migues [41]	2007	II	1		1						1			1		1	1	1	1		**8**
Neville [42]	2007	II																	1		**3**
Houck [43]	2008	II																	1		**3**
Krause [44]	2008	II	1			1					1		1			1			1		**6**
Wukich [45]	2008	II									1		1								**2**
Brodsky [46]	2009	II								1				1					1		**3**
Houck [47]	2009a	II																	1		**3**
Houck [48]	2009b	II																	1		**3**
Kohls-Gatzoulis [23]	2009	II								1	1	1		1	1	1			1		**7**
Neville [49]	2009	II																	1		**3**
Giorgini [50]	2010	II	1			1	1				1			1	1	1			1	1	**9**
Neville [51]	2010	II																	1		**3**
Parsons [52]	2010	II		1						1	1		1						1		**5**
Imai [53]	2011	II																			**5**
Brilhault [54]	2012	II	1							1			1								**3**
Kou [55]	2012	II	1								1		1	1		1	1		1		**7**
Neville [56]	2012	II																	1		**3**
Niki [57]	2012	II		1					1	1	1		1			1			1		**7**
Neville [58]	2013	II																	1		**3**
Chadwick [59]	2015	II		1				1		1	1		1			1			1		**7**
Houck [60]	2015	II											1						1		**3**
Neville [61]	2016	II																	1		**3**
Yoshioka [62]	2016	II									1		1						1		**4**
Silva [63]	2015	IIB		1							1		1			1			1		**5**
Kulig [64]	2009	I-II		1	1									1	1	1	1		1		**7**
Kulig [65]	2009	I-II		1	1					1											**3**
Kulig [66]	2011	I-II		1	1										1		1		1		**5**
Kulig [67]	2015	I-II		1	1										1		1		1		**5**
Weil [68]	1998	II-III	1		1	1	1	1		1			1			1			1		**9**
DiDomenico [69]	2011	II-III		1						1	1		1	1		1			1		**7**
Funk [6]	1986	NR			1		1				1					1					**4**
Chao [70]	1996	NR	1					1		1	1										**4**
Groshar [71]	1997	NR	1		1			1		1			1		1						**6**
Hsu [72]	1997	NR			1						1			1	1	1					**5**
Kitaoka [73]	1997	NR			1					1	1		1	1	1	1	1		1		**9**
Lim [74]	1997	NR	1	1							1		1			1					**5**
Stroud [75]	2000	NR		1					1		1		1								**4**
Augustin [76]	2003	NR					1				1			1		1					**4**
Kohls-Gatzoulis [77]	2004	NR								1	1			1	1	1					**5**
Alvarez [78]	2006	NR			1			1		1	1										**4**
Bulstra [79]	2006	NR		1	1	1		1													**4**
Satomi [80]	2008	NR		1					1				1	1	1	1					**6**
Sanhudo [81]	2014	NR	1	1																	**3**
Arnoldner [82]	2015	NR	1					1					1								**3**
Lin [83]	2015	NR	1	1									1								**3**
**AAFD**																					
Chimenti [84]	2014	II																	1		**3**
Spratley [85]	2014	IIB									1			1	1	1			1		**5**
Bolt [86]	2007	I-II											1	1	1	1	1		1		**6**
Jeng [87]	2011	IV								1	1			1	1						**4**
Harper [88]	1999	NR		1										1		1					**3**
Thomas [89]	2001	NR		1			1			1			1	1	1	1			1		**8**
Greisberg [90]	2003	NR		1									1								**2**
Kang [91]	2003	NR	1		1		1				1			1	1	1					**7**
Greisberg [92]	2005	NR		1									1								**2**
Arangio [93]	2006	NR									1			1	1	1		1			**5**
Arangio [94]	2006	NR													1	1					**2**
Arangio [95]	2009	NR		1							1			1	1	1		1			**6**
Kohls-Gatzoulis [23]	2009	NR												1	1	1					**3**

Legend: Black cells represent compulsory criteria and white cells represent not applicable to the individual article. Dark grey is a group of criteria relating to tendon dysfunction and light grey is a group of criteria relating to structural deformity where individual criteria in shaded boxes are optional and at least one from the group is compulsory.

Abbreviations: PTT: posterior tibial tendon; TOP: tender on palpation; res.: resisted; INV: inversion; SLHR: single leg heel raise; dec.: decreased; FF: forefoot; def.: deformity; HF: hindfoot; ABD: abduction; MF: midfoot; SUP: supination; Flex.: flexible; NR: not reported.

Twenty-four articles (24/82; 29.3%) did not report which stage of the condition the paper investigated (9 AAFD, 13 PTTD). Two articles (2/58; 3.4%) used Bluman’s classification system (1 AAFD, 1 PTTD) and the remaining 56 (56/58; 96.6%) used the Johnson and Strom classification. Of the 58 that did report stage of condition, 65.5% (38/58 articles) investigated stage II (1 AAFD, 37 PTTD). All articles investigating stage I dysfunction looked at PTTD (10/58; 17.2%). The remaining articles investigated stage I-II (5/58; 8.6%; 1 AAFD, 4 PTTD), IIB (2/58; 3.4%; 1 AAFD, 1 PTTD), II-III (2/58; 3.4%; 2 PTTD) and stage IV (1/58; 1.7%; 1 AAFD).

After collapsing variations in terminology (see Appendix 1), a total of 18 criteria were extracted from the 80 individual papers. The criteria were separated into two main groups; those pertaining to tendon dysfunction (10 criteria) and those relating to structural deformity (8 criteria). Those relating to dysfunction of the posterior tibial tendon were further categorised into symptoms of pain and swelling (7 criteria), and signs of deficits in strength or function (3 criteria). Pain was delineated based on location (i.e. along the posterior tibial tendon and/or medial ankle/foot) and provocating activity (i.e. tenderness on palpation, with resisted inversion and/or with single leg heel raise). Swelling was also separated into two categories based on location (i.e. along the posterior tibial tendon or the medial ankle/foot). The three criteria relating to strength or functional deficit were resisted inversion strength deficit, difficulty with single leg heel raise, and compromised walking ability. The eight criteria for foot posture and structural deformity were: flatfoot deformity, hindfoot valgus, medial arch collapse, forefoot abduction, midfoot abduction, forefoot supination, a flexible deformity, and talar head prominence.

Individual studies reported between 2 (5/82; 6.1%) and 9 (6/82; 7.32%) compulsory criteria for the diagnosis of PTTD or AAFD. The most frequently occurring number of compulsory criteria in any paper was 3 (25 articles; 30.49%), but specific criteria differed between articles.

The papers that referred to PTTD contained 69 diagnostic/inclusion criteria in 68 articles, with one paper (Kohls-Gatzoulis, 2009) describing criteria for both stage I and II PTTD separately (Table 2). Thirteen PTTD articles (18.8%) required symptoms of tendon dysfunction but not structural deformity; whereas, 56 articles (81.2%) required signs of both tendon dysfunction and structural deformity. Considering all listed signs and symptoms (n = 382), a flexible deformity (41; 10.7%), forefoot abduction (41; 10.7%) and difficulty with single leg heel raise (39; 10.2%) were the most frequently reported criteria (optional and compulsory) required for the diagnosis of PTTD.

**Table 2:** Frequency of criteria for diagnosis of PTTD and AAFD based on tendon symptoms, structural deformity and a combination of both.

	TENDON	STRUCTURE	BOTH	TOTAL
**AAFD**	0	2	11	13
**PTTD**	13	0	56	69
**TOTAL**	13	2	67	82

Thirteen papers describe diagnostic criteria for AAFD (Table 2). Eleven articles (84.6%) required both signs of tendon dysfunction and structural deformity. Two (15.4%) papers required only signs of structural deformity, with no mention of tendon dysfunction. Considering all signs and symptoms (n = 60), forefoot abduction (10; 16.7%), medial arch collapse (10; 16.7%) and hindfoot valgus (10; 16.7%) were most frequently reported criteria for the diagnosis of AAFD.

Considering all criteria for both PTTD and AAFD combined (n = 442), the most commonly reported (optional and compulsory) were forefoot abduction (51; 11.5%), a flexible deformity (45; 10.2%) and difficulty performing a single leg heel raise (44; 10.0%).

Considering signs and symptoms listed in articles investigating stage I PTTD (n = 36), the most frequently reported (optional and compulsory) criteria were tenderness on palpation of the posterior tibial tendon (6; 16.7%) followed equally by pain in the medial foot/ankle, swelling along the posterior tibial tendon, inversion strength deficit, and difficulty performing a single leg heel raise (4; 11.1%). There were no articles reporting grade 1 AAFD.

The most commonly reported criteria (n = 237) for stage II (including IIB) PTTD were the presence of a flexible deformity (33; 13.9%), forefoot abduction (28; 11.8%) and difficulty with single leg heel raise (43; 10.1%). There were 2 papers investigating stage II AAFD (including 2B) and the most frequently reported criteria were the presence of a flexible deformity, forefoot abduction, medial arch collapse and hindfoot valgus.

Consistent with data from when all PTTD studies were combined, when criteria (n = 293) for the early stages of PTTD were combined (stage I, II, I-II and IIB) the most frequently reported criteria were the presence of a flexible deformity (38; 13.0%), forefoot abduction (31; 10.6%) and difficulty with a single leg heel raise (28; 9.6%). When articles investigating the early stages of AAFD were combined, the most frequently reported criteria (n = 18) were the presence of a flexible deformity (3; 16.7%), forefoot abduction (3; 16.7%), medial arch collapse (3; 16.7%) and hindfoot valgus (3; 16.7%).

There were 2 articles describing stage II-III PTTD. Pain (either along the tendon, medial foot or with inversion or single leg heel raise), difficulty with resisted inversion, forefoot abduction and a flexible flatfoot deformity were reported in both studies. One article described diagnostic criteria for stage IV AAFD, which included difficulty with resisted inversion and single leg heel raise, hindfoot valgus and decreased medial longitudinal arch.

## Discussion

It is apparent from this systematic synthesis of available literature that there is significant overlap in the key signs and symptoms used to include PTTD and AAFD in research studies. While the hypothesis was to identify selection criteria, a major finding was that within the body of PTTD and AAFD literature, over half did not report how the condition was diagnosed. Of 228 primary research articles, 148 (65%) did not specify the specific criteria used to diagnose the condition or determine inclusion into the study (Fig 1). These studies frequently reported that the condition was diagnosed by the clinician or based on a classification system, without stating the impairments (signs and symptoms) that led to the diagnosis. Specifying impairments that confirmed diagnosis and led to inclusion in the study would improve consistency between studies and better enable comparisons. In order to appropriately apply evidence based practice in the clinic it is important to closely align or match patients with those reported in the literature. To accomplish this, it is essential that inclusion/diagnostic criteria are firstly reported in all studies and secondly consistent between studies. Of the articles investigating PTTD and AAFD that did report eligibility criteria, 67 (81%) included signs and symptoms relating to both tendon dysfunction and structural deformity. The exception to this was articles investigating early stage I PTTD where tendon signs (pain, swelling, weakness) were most prevalent. Although terminology for a tendon related condition was used (PTTD); the presence of signs and symptoms indicating an acquired flatfoot deformity were still required for a positive diagnosis and study inclusion for stage II and above PTTD. Similarly, articles using the terminology AAFD for the condition included signs and symptoms relating to dysfunction of the posterior tibial tendon, not just the acquired flatfoot deformity. This suggests, despite differing nomenclature, these articles are investigating the same condition, which is characterised by dysfunction of the posterior tibial tendon and an acquired flatfoot deformity.

When data for PTTD and AAFD were combined, the overarching diagnostic criteria were difficulty performing a single leg heel raise, the presence of a flexible deformity and forefoot abduction. This is consistent with early descriptions of PTTD and classification systems; in which the ‘too many toes’ sign (forefoot abduction), a flexible flatfoot deformity and difficulty inverting the calcaneus while rising onto the toes were reported as indicative of dysfunction of the posterior tibial tendon [6, 8].

**Fig 2:**
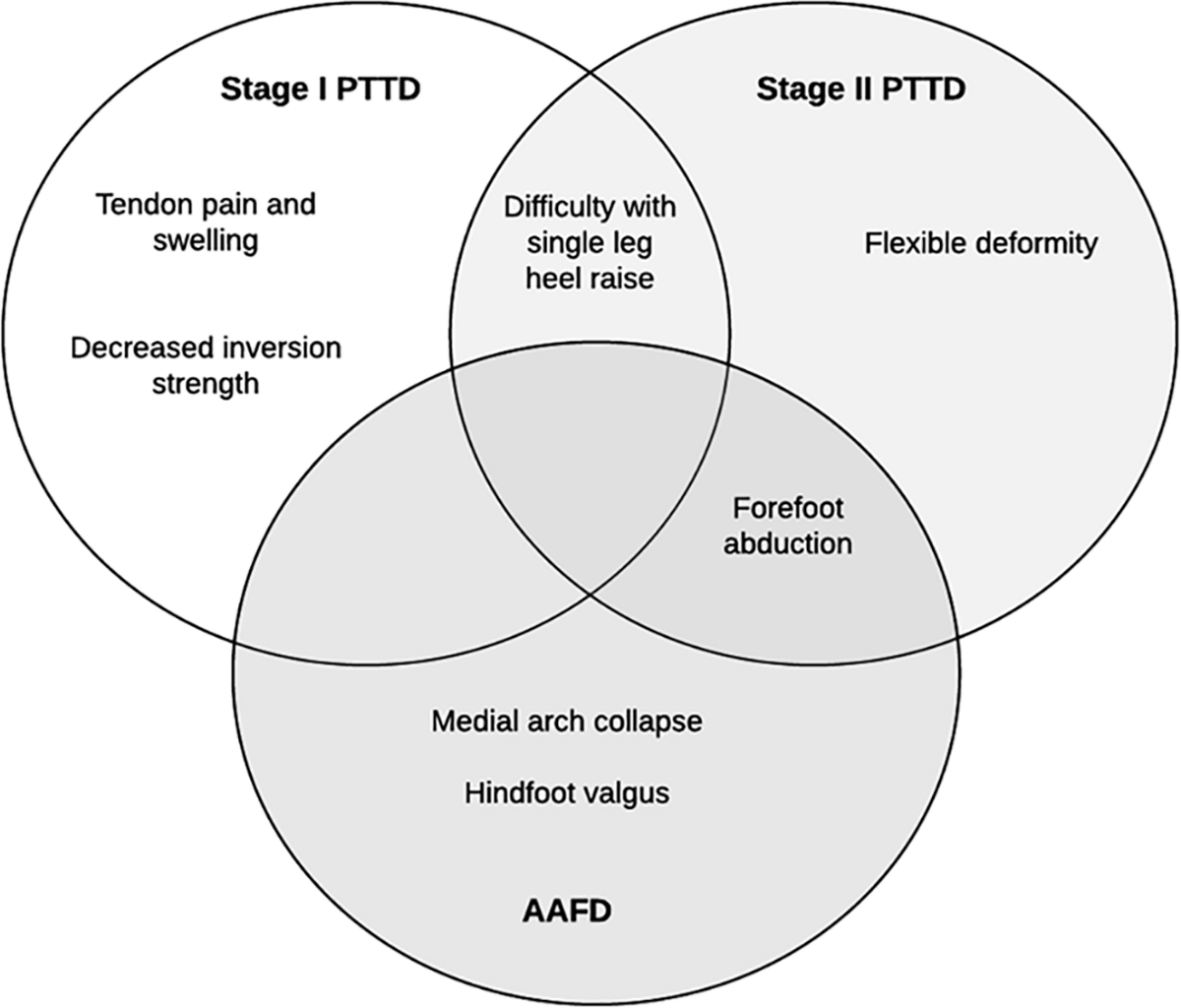
Diagrammatic summary. Similarities and differences in selection criteria for Stage I and II PTTD and AAFD.

Presenting signs and symptoms understandably vary with the stage of the condition. The majority of articles reported in this review pertain to stage I and or II PTTD and, consistent with the progressive nature of tendon dysfunction, there were clear differences between these stages. In stage I PTTD tenderness on palpation, pain and swelling around the tendon played a key role in diagnosis. These were not the most common diagnostic criteria for stage II PTTD. Tendon involvement was evidenced by impaired function (i.e. difficulty with single leg heel raise) rather than pain or inflammation, and diagnosis of this stage included signs of deformity (Fig 2). This suggests that in stage I the tendon is reactive [96], whereas in stage II it has progressed to a dysfunctional state where it is no longer able to invert the calcaneus and support the medial longitudinal arch.

There were commonalities in the criteria used to diagnose stage II PTTD and stage II AAFD. A flexible deformity and forefoot abduction were required for both diagnoses. Consistent with the nomenclature, tendon related symptoms (e.g. difficulty with single leg heel raise) were also required for the diagnosis of PTTD, whereas additional symptoms of structural deformity (e.g. hindfoot valgus and medial arch collapse) were required for the diagnosis of AAFD. An important consideration is that in the early stages (I-II) of both PTTD and AAFD, flexibility of the deformity is a key sign.

There were substantially less articles detailing diagnostic criteria for the later stages of PTTD and AAFD. Two articles described criteria for stage II-III PTTD. It is interesting that stage II and III were combined in these papers, as original classification systems have a clear delineation between stage II and III; being that the flatfoot deformity is flexible in stage II and rigid in stage III [8]. Both papers listed the presence of a flexible deformity as a key criteria, which suggests stage II was the condition being studied [68, 69]. The remaining criteria for these papers were also consistent with the most commonly reported signs for stage II PTTD. The criteria in the one paper investigating stage IV AAFD suggest that pain and inflammation are no longer key (or present) and the structural deformity and lasting functional deficits (e.g. difficulty with inversion and single leg heel raise) is emphasised. Two key issues have become apparent on review of the literature that did identify selection/inclusion criteria for PTTD and/or AAFD. First, PTTD and AAFD are being used interchangeably to describe the same condition. Where there are clear signs of a dysfunctional tendon (pain, swelling, weakness), we suggest the condition be referred to as PTTD. To negate the confusion surrounding early stages of the condition in which a flatfoot deformity is not present, we suggest that PTTD is the preferred terminology for the condition. The acquired flatfoot deformity may be a sign that develops in the later stages of the condition. This aligns with the literature that considers PTTD to be only one of several potential causes of AAFD [11, 14, 97].

Second, research studies use inconsistent inclusion criteria for participants with PTTD and AAFD. Based on data from studies included in this review, we recommended the following signs pertaining to tendon dysfunction form the inclusion criteria for studies investigating stage I PTTD: pain along the tendon, swelling and weakness with inversion and/or single leg heel raise. Suggested inclusion criteria for stage II include difficulty with single leg heel raise and a flexible flatfoot deformity; characterised by forefoot abduction, a lowered medial longitudinal arch and/or hindfoot eversion. Recommendations for stage III and IV are unable to be made as few studies investigated the later stages of the condition.

As PTTD is only one potential cause of AAFD, it is important to differentiate AAFD that is predominantly related to PTTD from other causes. An adult acquired flatfoot due to rheumatoid arthritis may not present with the same impairments (pain, function and/or disability) as those with an adult acquired flatfoot due to PTTD, nor will they likely respond in the same manner to conservation or surgical intervention. It is important to clearly characterise the key signs and symptoms of PTTD in isolation from other causes of AAFD in order to best guide effective treatment protocols. To avoid potential misunderstanding, it stands to reason that when AAFD is used in the literature as an umbrella term for acquired flatfoot deformities, the underlying aetiology of the AAFD should be reported. As there are considerable differences in the diagnostic criteria used in each stage of PTTD and AAFD, it is also important that the stage of the condition be indicated.

There are some limitations that need to be considered for this review. First, due to resource implications, after the search strategy was developed, a single reviewer independently searched the literature and assessed eligibility. Secondly, a hand search was not employed due to the broad search terms used and the large number of references retrieved. Thirdly, we might have excluded some studies that only stated they used a classification system and did not list the specific selection criteria. We felt justified in doing this to avoiding ambiguity in matching our extracted data and that which was specifically reported in those papers.

In conclusion, it is recommended that PTTD is the preferred terminology for the condition of a painful, dysfunctional posterior tibial tendon, even in the later stages where an acquired flatfoot deformity has developed. This will remove ambiguity regarding other potential causes for AAFD. There is a need for more consistent and uniform reporting of inclusion/selection criteria for studies investigating PTTD. This article had outlined suggested eligibility criteria for stages I and II of the condition that can be used in future research and will enhance the applicability of evidence based practice in the clinic.

## Supporting information

S1 Checklist PRISMA checklist. (DOC)

S1 Table Categories used in Table 1. (PDF)
