# Supplementary material for: Reported selection criteria for adult acquired flatfoot deformity and posterior tibial tendon dysfunction: Are they one and the same? A systematic review
Source: PLoS One. 2017 Dec 1;12(12):e0187201. doi: 10.1371/journal.pone.0187201 (PMC5711021; doi:10.1371/journal.pone.0187201)
Supplement: S1 Table — (PDF) [file pone.0187201.s002.pdf]

S1 Table. Categories used in table 1

Headings	Terms used in study inclusion and diagnostic criteria
Pain along PTT	Pain over the tendon
Pain medial ankle/foot	Posteromedial ankle pain, medial hindfoot pain, pain on medial aspect of arch of the foot, medial ankle pain
Tender on palpation PTT	Palpable tenderness, tenderness over the tendon, pain on palpation of the PTT, tenderness along PTT, tenderness to palpation, pain reproduced on palpation of the PTT
Pain with resisted inversion	
Pain with SLHR	Pain with unilateral heel rise, pain when rising onto toes while weightbearing,
Swelling along PTT	Localized oedema, swelling over the tendon, local swelling, swelling along the course of PTT, swelling over tendon sheath, enlargement over the posterior tibial tendon,
Swelling medial ankle/foot	Medial hindfoot swelling, swelling posteromedial aspect of ankle, swelling on medial aspect of arch of the foot, enlargement of the medial retromalleolar region,
Inversion strength deficit	Weakened inversion force, weakness of posterior tibialis, positive resistance test results, reduced or absent power of inversion, clinical strength deficit when tested with the foot in inversion and plantarflexion, weak strength upon supination on manual testing, inability to bring the foot across the midline from an abducted position, diminished power PT
Difficulty with SLHR	Inability to perform SLHR, lack of active hindfoot inversion during SLHR, inability to do single-limb toe raises, difficulty lifting one foot
Decreased walking ability	Difficulty ambulating, decreased ability to walk any distance, reduced walking distance
Flatfoot deformity	Acquired flatfoot deformity, planovalgus deformity, pes planovalgus
Hindfoot valgus	Heel valgus, rearfoot abduction, calcaneal valgus, hindfoot eversion, valgus angulation of the heel
Medial arch collapse	Flattening of medial longitudinal arch, midfoot collapse, loss of height in the arch, loss of longitudinal arch, loss in medial arch contour, arch collapse, flattened midfoot posture, fallen medial longitudinal arch
Forefoot abduction	Too many toes sign, varus forefoot, first metatarsal abduction, lateral deviation of the forefoot
Midfoot abduction	Abduction at the transverse tarsal joint, abducted midfoot posture
Forefoot supination	
Flexible deformity	Passively correctable deformity, flatfoot deformity with a mobile hindfoot, hindfoot valgus passively correctable, flexible pes planovalgus, manually correctable valgus deformity, non-fixed hindfoot valgus deformity, supple deformity, absence of rigid foot deformity, mobile mid- and hind-foot
Talar head prominence	
